# Effects of a Neuroscience-Based Mindfulness Meditation Program on Psychological Health: Pilot Randomized Controlled Trial

**DOI:** 10.2196/40135

**Published:** 2023-01-19

**Authors:** Sarah Lynn, Julia C Basso

**Affiliations:** 1 Department of Human Nutrition, Foods and Exercise Virginia Tech Blacksburg, VA United States; 2 School of Neuroscience Virginia Tech Blacksburg, VA United States; 3 Center for Health Behaviors Research Fralin Biomedical Research Institute at Virginia Tech Roanoke, VA United States

**Keywords:** meditation, mindfulness, mental health, compassion, self-compassion, digital, medical education, neuroscience education, depression, psychological health, mental illness, anxiety

## Abstract

**Background:**

Mindfulness and meditation have a rich historical tradition, and a growing scientific base of evidence supports their use in creating positive psychological and neuroplastic changes for practitioners. Although meditation can be taught in various ways, the scientific community has yet to systematically study the impact of different types of meditation on neuropsychological outcomes, especially as it pertains to digital implementation. Therefore, it is critical that the instruction of mindfulness be evidence based because meditation is being used in both scientific and clinical settings.

**Objective:**

This study investigated the use of teacher cueing and the integration of neuroscience education into a meditation program. Compassion cueing was chosen as the element of experimental manipulation because traditional lineages of Buddhist meditation teach compassion for self and others as one of the primary outcomes of meditation. We hypothesized that participants receiving compassion cueing would have enhanced neuropsychological outcomes compared with those receiving functional cueing and that gains in neuroscience knowledge would relate to positive neuropsychological outcomes.

**Methods:**

Participants (n=89) were randomized to receive either functional cueing (control group) or compassion cueing (experimental group) and engaged with five 10-minute meditation sessions a week for 4 weeks. All intervention sessions were administered through digital presentation. All participants completed ecological momentary assessments before and after the daily intervention, as well as pre- and postintervention questionnaires.

**Results:**

Participants demonstrated significant benefits over time, including increased mindfulness and self-compassion, decreased depression, and gains in neuroscience content (all *P*<.001); however, no significant between-group differences were found. Daily scores from each day of the intervention showed a statistically significant shift from active toward settled. Importantly, long-term increases in mindfulness were positively correlated to changes in compassion (*r*=0.326; *P*=.009) and self-compassion (*r*=0.424; *P*<.001) and negatively correlated to changes in anxiety (*r*=–0.266; *P*=.03) and depression (*r*=–0.271; *P*=.03). Finally, the acute effects of meditation were significantly correlated to the longitudinal outcomes (with a small-to-medium effect size), especially those relevant to mindfulness.

**Conclusions:**

We developed a novel neuroscience-based education–meditation program that enhanced self-regulation as evidenced by improved mindfulness, self-compassion, and mood state. Our findings demonstrate the behavioral importance of engaging with mindfulness meditation and reinforce the idea that the benefits of meditation are independent of teacher cueing behavior. Future studies will need to investigate the brain-based changes underlying these meditation-induced outcomes.

## Introduction

### Background

Mindfulness and meditation have a rich historical evidence base, originating in Asia as part of Buddhist and yogic traditions [1]. The yoga sutras of Patanjali, a collection of Sanskrit statements on the theory and practice of yoga, define mindfulness as the awareness of self [2]. In contemporary Buddhism, Bodhi [3] traces the history of mindfulness back to the Pali canon (written in the first century BCE), a canonical text foundational to Buddhist traditions. A modern interpretation of the Pali canon describes mindfulness as “lucid awareness.” In the 1970s, Kabat-Zinn [4], the founder of the Center for Mindfulness at the University of Massachusetts Memorial Medical Center, defined mindfulness as “...the awareness that emerges through paying attention on purpose, in the present moment, and nonjudgmentally to the unfolding of experience moment by moment.” This *modern* definition of mindfulness separates spirituality from the established baseline set of characteristics used to define the psychological state of mindfulness (eg, paying attention to the present moment) [1].

Meditation is a self-regulatory practice of the body and mind that can be performed in various ways and is a nonpharmaceutical low-cost tool that can be integrated into a healthy lifestyle [5]. A host of literature has examined the effects of meditation on a range of psychological factors and brain outcomes, with multiple systematic reviews and meta-analyses published in this field [6-9]. Meditation substantially promotes the psychological outcome of mindfulness [10]. Furthermore, meditation decreases symptoms of anxiety and depression [11,12]; improves executive functioning, including attention [13]; and enhances self-compassion [14]. Although meditation is often examined in healthy populations, the benefits of meditation are currently being studied in specific clinical populations such as those with psychiatric illness and substance use disorder [15]. Various studies have used neuroimaging techniques to probe the neural mechanisms of meditation, finding statistically significant increases in activation of the insula, a brain region involved in self-awareness, empathy, and interoception [16]. The literature also supports decreased amygdala activation paired with increased anterior cingulate cortex and prefrontal cortex activation, indicating that meditation may provide effects at both the bottom-up and top-down levels [10,17]. Researchers theorize that these meditation-induced neural changes may support psychological outcomes, including increased present-moment focus and decreased self-referential thinking or rumination [17]. In addition, meditation has been shown to alter resting state functional connectivity in the default mode and dorsal attention networks, which is suggested to contribute to the clinical benefits of meditation, including decreases in depression and anxiety [18,19]. Although the size of the scientific literature supporting the benefits of meditation is increasing, it is currently unknown how specific teaching practices within meditation may augment the psychological outcomes.

The use of mindful meditation is not standardized [20], and no professional or regulatory body exists to support teaching or implementing mindfulness interventions in a scientific or clinical setting [21]. Farias and Wikholm [21] highlight the multitude of mental and physical outcomes of mindfulness [22,23] but note that to optimize outcomes, physical and mental health practitioners will require specific training and certification in mindfulness techniques. It is recommended that instructors of mindful meditation have embodied experience with the practice [24]; however, translating personal experiences into clinical interventions of mindfulness requires careful consideration. Lessons learned from a clinical experience in the United Kingdom outline six domains of teacher competence: (1) coverage and pacing of session curriculum, (2) relational skills, (3) guiding mindfulness practices, (4) conveying course themes through teaching, (5) embodiment of mindfulness, and (6) management of group process [25]. In their handbook, *Resources for Teaching Mindfulness*, McCown et al [26] suggest that scientific literacy is also a foundational competency for teachers of mindful meditation [26]. However, it is unknown whether scientific literacy about mindful meditation changes participant outcomes.

### Objectives

In this single-blind randomized controlled trial (RCT), we explored how teachers of mindfulness use their words and curriculum to guide and instruct meditation to optimize psychological outcomes, including mindfulness, compassion, self-compassion, and mental health. Although meditation can be taught in many different ways, we chose compassion cueing as the element to manipulate because traditional lineages of Buddhist meditation teach compassion for self and others as one of the primary outcomes of meditation [27,28]. In addition, this intentionally scaffolded 4-week meditation program was designed within the framework of a neuroscience education curriculum. All participants learned about the neuroscience of meditation, with a special focus on attentional control, emotional regulation, self-awareness, and self-regulation, before engaging in the actual practice of meditation. We hypothesized that there would be a statistically significant correlation between the gains in neuroscience knowledge and our psychological outcomes of interest. Before and after meditation, daily check-ins provided information about the acute effects of meditation on thoughts, emotions, and bodily sensations. We hypothesized that larger acute gains would correlate to larger long-term gains from meditation, which is yet another unexplored area of research. Understanding the impact of the curricular implementation of meditation will help to fill the gap in best practices and competencies for inclusion in future mindfulness interventions.

## Methods

### Participants

To be eligible for the study, participants were required to be aged ≥18 years and be fluent in English. The exclusion criteria were active trauma or diagnosed and untreated psychiatric illness, which were both self-reported through an initial screening questionnaire developed by the investigators. Recruitment was conducted using social media platforms, other web-based forums, and through in-person announcements by Virginia Tech faculty members who were teaching related content (ie, mindfulness and meditation). The recruitment language included an invitation to practice meditation in the participant’s own home, learn the neuroscience supporting mindfulness meditation, and contribute to new scientific discoveries. Participants were compensated up to US $25 for their participation, with proration occurring at US $2.50 for the pretest assessment, US $1 for each day of meditation, and US $2.50 for the posttest assessment.

### Ethics Approval

The institutional review board at Virginia Tech reviewed and approved this study (IRB-20-799), and all participants completed informed consent.

### Study Design

This study was a 4-week RCT, with participants randomized to receive either meditation with functional cues (control group) or meditation with self-compassion cues (experimental group). Participants were randomly assigned to the groups through a web-based random number generator. Participants completed pre- and posttest questionnaires along with daily acute assessments. This RCT was partially blinded because the investigators knew participant group assignment; however, participants were unaware of their group assignment. The study included 10-minute meditation sessions 5 days a week. Each day, participants were instructed to watch a 10-minute prerecorded education-meditation video. The control group received standard functional meditation directions (ie, “If you are distracted, return to the mantra.”), whereas the experimental group received functional meditation directions and additional self-compassion cues (ie, “When you are distracted, remember that’s okay. Try to return to the mantra.”). Both groups received identical neuroscience education portions of this study. Participants engaged in neuropsychological assessments before and after the 4-week intervention. In addition, participants completed momentary assessments before and after the presentation of the daily education-meditation video.

The weekly schedule included a scaffolded curriculum and was the same for each of the 4 weeks. Day 1 included 7 minutes of neuroscience education and 3 minutes of meditation. Days 2, 3, and 4 included 5 minutes of neuroscience education and 5 minutes of meditation. The final day of the week was a 10-minute meditation practice (Table 1). The scaffolding of the meditation experience was specifically designed to develop meditation skills over the course of the week, building toward the culminating 10-minute practice; this design is also an example of the flipped classroom model [29].

The education-meditation curriculum was designed by a PhD neuroscientist and an experienced meditation teacher with >10,000 hours of teaching experience. The first week of the curriculum focused on attentional control and used focused attention meditation. Focused attention meditation used the mantra or repeated phrase, “I am alive. I am at ease.” The second week of the curriculum focused on emotional control and used an open monitoring meditation. This technique, called *movie of the mind*, instructed participants to watch their thoughts without engaging with, or trying to change, them. The third week of the curriculum focused on self-awareness and used the practice of embodiment or connecting mind and body through intentional movement with awareness. Hand turning paired with the breath facilitated mindful embodiment as participants were guided to feel and respond to bodily sensations. The fourth week of the curriculum focused on self-regulation and used the practice of breath control. Participants were instructed to observe their breath without interrupting it (Table 2).

**Table 1:** Weekly schedule for the division of time spent in the intervention. This schedule was repeated 4 times, with a new meditation technique provided weekly.

Day	Minute									
	1	2	3	4	5	6	7	8	9	10
1	Neuroscience education	Neuroscience education	Neuroscience education	Neuroscience education	Neuroscience education	Neuroscience education	Neuroscience education	Meditation	Meditation	Meditation
2	Neuroscience education	Neuroscience education	Neuroscience education	Neuroscience education	Neuroscience education	Meditation	Meditation	Meditation	Meditation	Meditation
3	Neuroscience education	Neuroscience education	Neuroscience education	Neuroscience education	Neuroscience education	Meditation	Meditation	Meditation	Meditation	Meditation
4	Neuroscience education	Neuroscience education	Neuroscience education	Neuroscience education	Neuroscience education	Meditation	Meditation	Meditation	Meditation	Meditation
5	Meditation	Meditation	Meditation	Meditation	Meditation	Meditation	Meditation	Meditation	Meditation	Meditation

**Table 2:** How neuroscience concepts, meditation style, and meditation techniques were related and utilized in this study design.

	Week 1	Week 2	Week 3	Week 4
Neuroscience^a^	Attentional control	Emotional regulation	Self-awareness	Self-regulation
Meditation style^b^	Focused attention	Open monitoring	Embodiment	Breath control
Meditation technique	Mantra	Movie of the mind	Hand turning	Observing the breath

^a^All groups received the same neuroscience curriculum.

^b^During meditation, the control group received functional cues, and the experimental group received functional cues plus compassion cues.

Regarding the weekly flow of the experiment, participants received an email (on a Sunday) with a link to begin their education-meditation program on the following day. Clicking on the provided link brought the participants to a Qualtrics survey with 3 daily questions:

- “How are your thoughts?”
- “How are your feelings?”
- “How is your body?”

The questions were answered on a standard 10-point Likert scale ranging from 1=“Settled*”* to 10=*“*Active.” This set of questions, although not validated, showed sensitivity to change in response to daily interventions. These questions were followed by a prerecorded video with neuroscience education content and lightly guided meditation, followed by a repeat of the daily questions. Once the second set of daily questions was complete, Qualtrics automatically sent the next day’s link 12 hours later. This system allowed participants flexibility to fit their meditations into the most convenient and reliable time during their day. If participants missed a session, reminder emails were sent to them to complete the sessions over the weekend to enable them to complete 5 sessions every 7 days.

Time spent watching the 10-minute video was monitored in Qualtrics with a clock feature on the embedded video page: <10 minutes meant that the participants did not complete the session, and >10 minutes suggested that they may have been distracted with other tasks or fallen asleep. Participants displaying inconsistent times more than once (3/89, 3%) were removed from the study.

### Study Measures

#### Beck Anxiety Inventory

The Beck Anxiety Inventory (BAI) is a self-report measure of anxiety symptoms [30]. Questions about somatic and psychological experiences related to anxiety are included. The BAI includes 21 items for reflection on a 4-point Likert scale, with 0 indicating “Not at all” and 3 indicating “It bothered me a lot.” The total score is calculated by summing responses for each question. Results range from 0 to 21=low anxiety, 22 to 35=moderate anxiety, and ≥36=potentially concerning anxiety levels. The BAI demonstrates high internal consistency, with Cronbach α=.91 and median item correlations at *r*=0.56. Principal components analysis with eigenvalues >1.0 with a varimax rotation converged in 19 iterations resulted in 5 factors, which accounted for 60% of the variance.

#### Beck Depression Inventory

The Beck Depression Inventory (BDI) is a self-report measure of depression symptoms, with >25 years of validity testing [31]. The BDI has 21 items, presented in a multiple-choice response format, with the participant selecting the phrase that best reflects their response to the prompt (eg, “I do not feel sad,” “I feel sad,” “I am sad all the time, and I can’t snap out of it,” or “I am so sad and unhappy that I can’t stand it”). Each possible response has an assigned value from 0 to 3, summed for a total score. The sums are then rated as follows: 1 to 10=these ups and downs are considered normal, 11 to 16=mild mood disturbance, 17 to 20=borderline clinical depression, 21 to 30=moderate depression, 31 to 40=severe depression, and >40=extreme depression. The reliability of the BDI was tested in clinical (Cronbach α=.86) and nonclinical (Cronbach α=.80) populations. The test-retest reliability demonstrated *r*>0.60. Concurrent validity with the Hamilton Rating Scale for Depression showed *r*=0.72 to 0.73 for clinical populations and *r*=0.60 to 0.74 for nonclinical populations [32].

#### Five-Facet Mindfulness Questionnaire

The Five-Facet Mindfulness Questionnaire (FFMQ) is a self-report measure of trait mindfulness behaviors and mindful thought patterns (Baer et al [33]). It is valid and reliable, demonstrating Cronbach α values ranging from .72 to .92. Confirmatory factor analysis with principal axis factoring with oblique rotation along with scree plots suggests a 5-factor structure. The FFMQ has 39 items and uses a 5-point Likert scale, with 1 representing “Never or very rarely true” and 5 representing “Very often or always true.” The 5 subscales include observing, describing, acting with awareness, nonreacting, and nonjudging. Scoring uses the total sum and sums of subscales, with specific questions scored in reverse.

#### Mindful Attention Awareness Scale

The Mindful Attention Awareness Scale (MAAS) measures core qualities of mindfulness and consciousness [34]. The MAAS consists of 15 items without subscales. Items are scored by individuals on a 6-point Likert scale, with 1 representing “Almost always” and 6 representing “Almost never.” The MAAS was tested in various populations, with Cronbach α values ranging from .80 to .87; after final modifications, the internal consistency of the 15 items showed a Cronbach α value of .82. In a general adult population, the Cronbach α value was .87. Known group validity showed sensitivity to Zen meditators, clinical populations, and the general adult population.

#### Self-Compassion Scale

The Self-Compassion Scale (SCS) was created by Neff [35] as a self-report measure of kindness and understanding toward oneself during times of struggle. The SCS is a 26-item scale measured on a 5-point Likert scale, with 1 representing “Almost never” and 5 representing “Almost always.” The SCS has 6 subscales: self-kindness, self-judgment (reverse scored), common humanity, isolation (reverse scored), mindfulness, and overidentification (reverse scored). Scoring is completed by calculating the means of each subscale, reversing where indicated, and then taking the sum of the subscale means. Cronbach α (.92) shows high internal consistency, with Cronbach α values ranging from .75 to .81 for each of the 6 subscales. Test-retest reliability is high (*r*=0.93), with subscale values ranging from *r*=0.80 to *r*=0.88. Exploratory structural equation modeling found a good fit with 6 factors, with 95% of the variance being attributed to the general factor.

#### Compassion Scale

The Compassion Scale (CS) is a self-report measure of one’s kindness and desire to lessen the struggle of others [36]. The CS includes 16 items divided among 4 subscales: kindness, common humanity, mindfulness, and indifference (reverse scored). The overall score is a total mean. Subscales are also represented as means. A variety of studies show the CS to be reliable, with Cronbach α values ranging from .77 to .90. Test-retest reliability demonstrated *r*=0.81. Known group validity showed marked differences, as expected in meditators versus nonmeditators. Structural equation modeling found a good fit with 3 positive subscales and 1 negative subscale.

#### Neuroscience Knowledge Check

To assess neuroscience knowledge, we created the Neuroscience Knowledge Check (NKC) to partner with content presented in the education portion of this study (Multimedia Appendix 1). This knowledge check helps to determine whether neuroscience knowledge acquisition influences mental health, compassion, and mindfulness outcomes. The NKC consists of 50 items, with 4 subscales paired with one of each of the 4 meditation techniques. Each question offered 4 options: 2 incorrect answers, 1 correct answer, and a selection for “I don’t know.” The inclusion of “I don’t know” as an answer offered participants an honest way to report what they know rather than having participants guess at the correct answer. The total score is a sum of the number of correct answers.

### Power and Statistical Analysis

An a priori power analysis was run using G*Power (version 3.1; Heinrich Heine University) to determine the appropriate number of participants to power this study. We used an *F* test, repeated measures ANOVA, within-between interaction using an effect size of 0.25, an α error probability of .0001 to correct for multiple testing, a power level of 0.8, 2 groups (functional cueing vs compassion cueing), 2 measurements (before the intervention vs after the intervention), correlation among repeated measures of 0.5, and a nonsphericity correction epsilon of 1 to determine a sample size of n=98.

Repeated measures ANOVAs with within-between interactions were conducted to examine the hypothesis that teacher behavior (ie, compassion cueing) significantly enhanced mindfulness (ie, FFMQ and MAAS), compassion (ie, CS and SCS), and mental health (ie, BAI and BDI) outcomes in the experimental group compared with the control group. We additionally used Pearson product-moment correlations to test the hypothesis that greater gains in neuroscience knowledge (ie, NKC) would be significantly associated with greater changes in mindfulness, compassion, and mental health outcomes and further that these outcomes of interest would be significantly correlated to one another. Paired samples 2-tailed *t* tests were used to assess the acute effects of meditation from before to after each session. Repeated measures ANOVAs were used to determine differences among the types of acute changes (ie, thoughts, emotions, and body) across the 20 days of the intervention. An α value of *P*<.05 was used to determine statistical significance. To correct for multiple testing in a family of analyses, Bonferroni corrections were used as appropriate. SPSS software (version 27.0; IBM Corp) was used for all statistical analyses. Because of technical issues in randomization procedures (ie, Qualtrics was programmed to present 6 rather than 7 questionnaires), participants received 6 of 7 postintervention neuropsychological assessments, resulting in different numbers of participant responses reported in the results.

## Results

### Participants

Of the 89 participants in this study, 65 (73%) were women, 67 (75%) identified as White, and 52 (58%) were novice meditators. The participants represent diverse annual income, education, and marital status (Table 3). Of the original 127 participants recruited for the study, 35 (39%) dropped out of the study before completion of the posttest assessment, and 3 (3%) were removed for lack of fidelity to research protocols, leaving a total of 89 participants.

**Table 3:** Demographic data for all participants (N=89).

Category			Participants, n (%)
**Sex**			
	Female	65 (73)	
	Male	23 (26)	
	Intersex	1 (1)	
**Race**			
	Asian	13 (15)	
	Black or African American	5 (6)	
	Other	4 (4)	
	White	67 (75)	
**Ethnicity**			
	Hispanic	2 (2)	
	Non-Hispanic	87 (98)	
**Education**			
	Advanced degree	65 (73)	
	Bachelor’s degree	19 (21)	
	Some college	4 (4)	
	Finished high school	1 (1)	
**Meditation experience**			
	None	14 (16)	
	Novice	52 (58)	
	Intermediate	20 (22)	
	Advanced	2 (2)	
	Expert	1 (1)	
**Marital status**			
	Divorced	8 (9)	
	Living with significant other	10 (11)	
	Married	44 (49)	
	Single (never married)	26 (29)	
	Widow or widower	1 (1)	
**Annual income (US $)**			
	<15,000	3 (3)	
	15,000 to 24,999	11 (12)	
	25,000 to 34,999	7 (8)	
	35,000 to 49,999	14 (16)	
	50,000 to 74,999	6 (7)	
	75,000 to 99,999	14 (16)	
	100,000 to 149,000	14 (16)	
	150,000 to 199,999	8 (9)	
	≥200,000	11 (12)	
**Employment status**			
	Homemaker	4 (4)	
	Not working	7 (8)	
	Retired	13 (15)	
	Working full time	46 (52)	
	Working part time	19 (21)	

### Study Measures Assessed Before and After the Intervention

**Figure 1:**
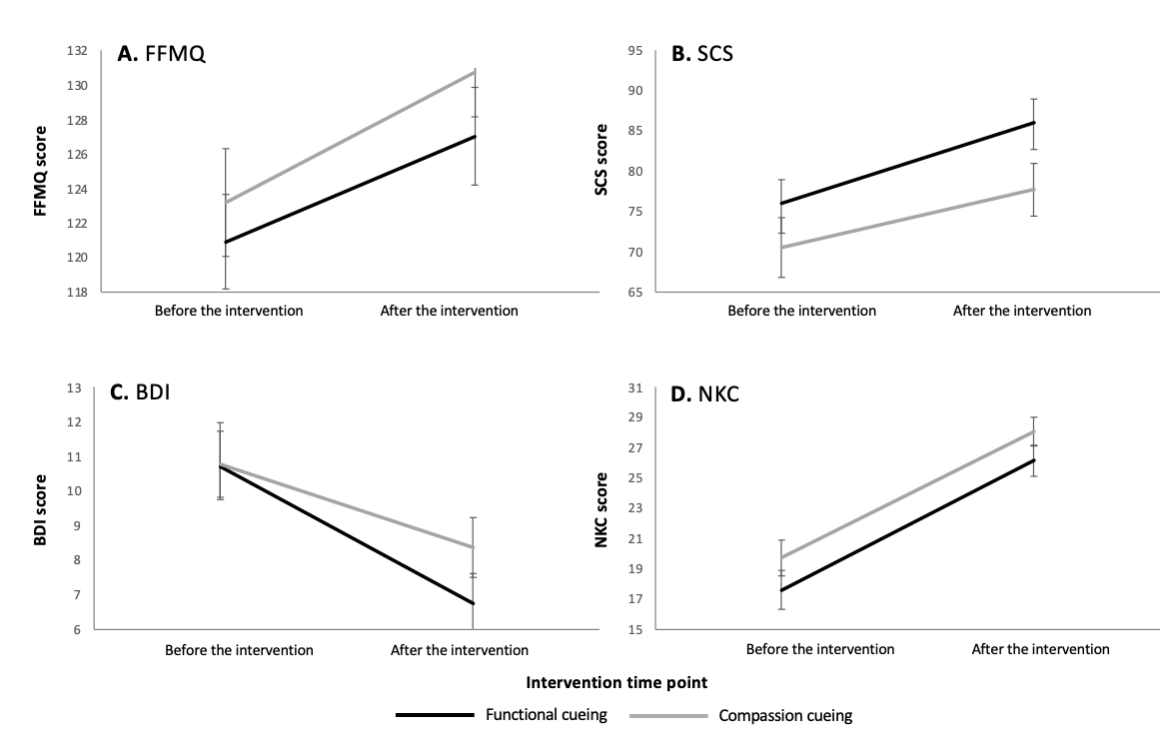
Pre- and posttest measurements for groups receiving either functional cueing or compassion cueing (n=89 for all tests). The education-meditation program significantly improved (A) mindfulness, (B) self-compassion, (C) depression, and (D) neuroscience knowledge. All time effects are significant at *P*<.001. BDI: Beck Depression Inventory; FFMQ: Five-Facet Mindfulness Questionnaire; NKC: Neuroscience Knowledge Check; SCS: Self-Compassion Scale.

No significant interaction (time × group) effect was found for any of our primary outcomes of interest (Multimedia Appendix 2). However, a significant time effect was found for mindfulness (Figure 1A; FFMQ: *F*_1,75_=26.595; *P*<.001; partial η^2^=0.262), with mindfulness scores significantly increasing from before the intervention to after the intervention. This effect was driven by the FFMQ subscales of observing (*F*_1,75_=12.680; *P*<.001; partial η^2^=0.145), describing (*F*_1,75_=10.566; *P*=.002; partial η^2^=0.123), acting with awareness (*F*_1,75_=5.706; *P*=.02; partial η^2^=0.071), nonjudging (*F*_1,75_=8.108; *P*=.006; partial η^2^=0.098), and nonreactivity (*F*_1,75_=24.622; *P*<.001; partial η^2^=0.247).

In addition, a significant time effect was found for self-compassion (Figure 1B; SCS: *F*_1,70_=26.406; *P*<.001; partial η^2^=0.274), with self-compassion scores significantly increasing from before the intervention to after the intervention. This effect was driven by the SCS subscales of self-kindness (*F*_1,70_=15.942; *P*<.001; partial η^2^=0.185), self-judgment (*F*_1,70_=5.149; *P*=.03; partial η^2^=0.069), isolation (*F*_1,70_=14.091; *P*<.001; partial η^2^=0.168), mindfulness (*F*_1,70_=16.070; *P*<.001; partial η^2^=0.187), and overidentification (*F*_1,70_=169.324; *P*<.001; partial η^2^=0.708).

A significant time effect was also found for depression (Figure 1C; BDI: *F*_1,78_=32.852; *P*<.001; partial η^2^=0.296), with depression scores significantly decreasing from before the intervention to after the intervention.

Finally, a significant time effect was found for neuroscience knowledge (Figure 1D; NKC: *F*_1,87_=126.387; *P*<.001; partial η^2^=0.592), with scores increasing from before the intervention to after the intervention.

Neither time effects nor time × group effects were significant for anxiety (BAI), compassion (CS), or dispositional mindfulness (MAAS; Multimedia Appendix 2).

### Study Measures Assessed Before and After the Daily Education-Meditation Practice

**Figure 2:**
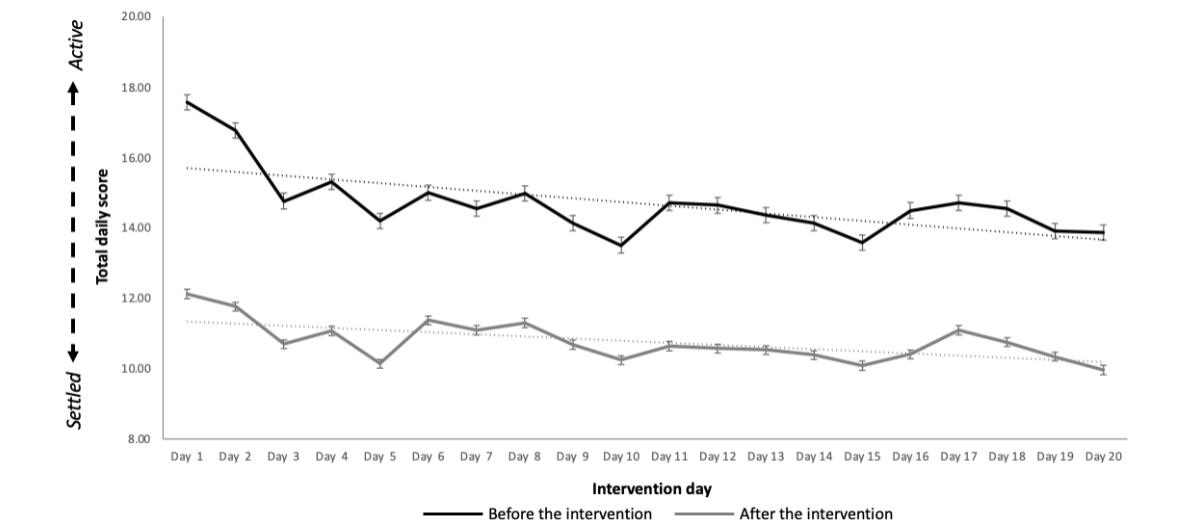
Mean (SE of the mean) of the total daily score, combining thoughts, emotions, and body from settled to active both before (black line) and after (gray line) watching the daily education-meditation video. All values are significant at *P*<.001.

As no significant time × group differences were established for measurements assessed before and after the intervention, the following data were analyzed across groups. Daily scores were significantly lower after the meditation than those before the meditation on all days (*P*<.001), demonstrating a significant acute effect of the daily education-meditation practice from a state of active toward settled (Figure 2; Multimedia Appendix 3).

**Figure 3:**
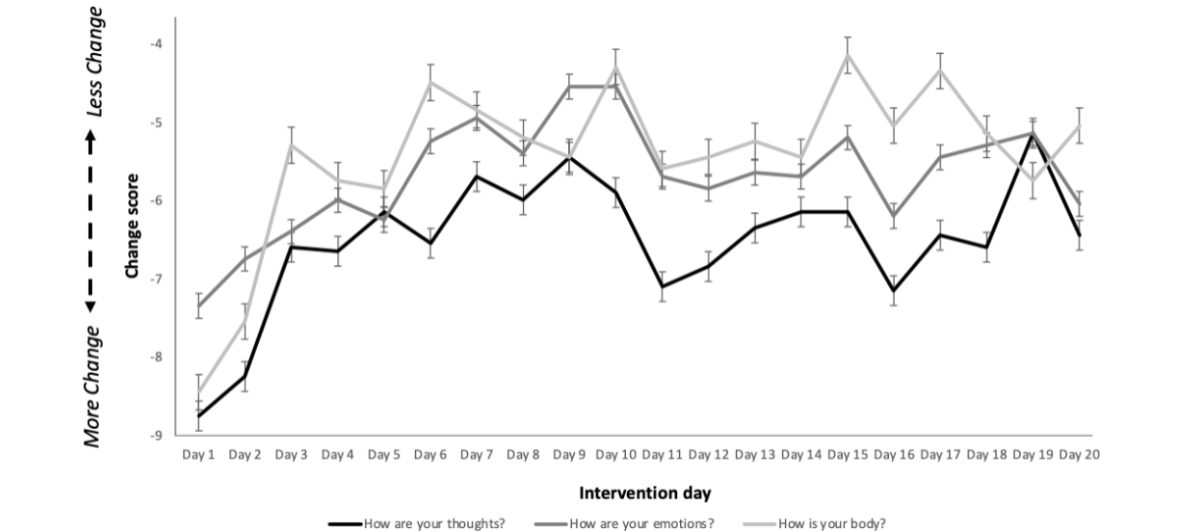
Absolute change scores, represented as mean (SE of the mean), for aspects of thoughts, emotions, and body from before to after the education-meditation practice. Larger values represent larger shifts in the direction from active to settled.

Change scores from before to after the daily education-meditation practice demonstrated a statistically significant effect over the 20 days of the intervention (Figure 3; *F*_15.044,4005.997_=5.140; *P*<.001; partial η^2^=0.019); however, no time × type (ie, thoughts, emotions, and body) differences were found (*F*_30.007,4005.997_=0.573; *P*=.97; partial η^2^=0.004).

**Figure 4:**
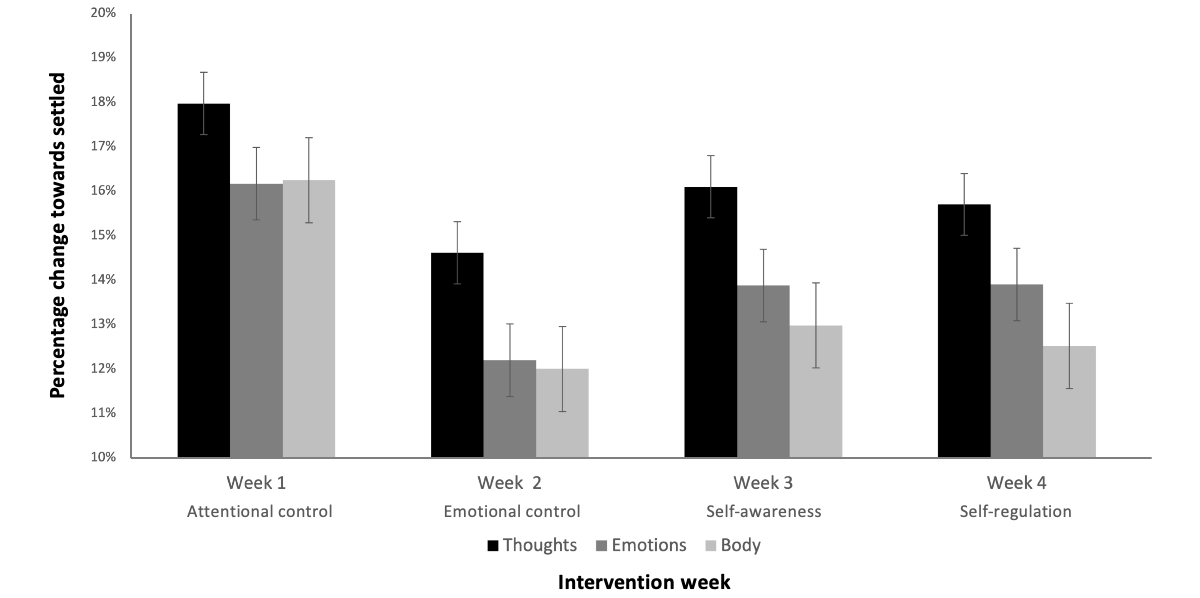
Percentage change towards settled from before to after the education-meditation practice for thoughts, emotions, and body during each week of the intervention.

As the intervention provided a unique meditation each week, we additionally visualized and analyzed the data on a per-week basis (Figure 4). When each week was analyzed independently, no between-group effects were found for week 1 (*F*_2,267_=2.272; *P*=.11), week 2 (*F*_2,267_=1.520; *P*=.22), week 3 (*F*_2,267_=2.026; *P*=.13), or week 4 (*F*_2,267_=1.910; *P*=.15). However, when data were analyzed across weeks, both time effects (*F*_2.678,715.034_=3.389; *P*=.02; partial η^2^=0.013) and time × type effects (*F*_5.356,715.034_=5.606; *P*<.001; partial η^2^=0.040) were found. Post hoc analyses revealed that although the acute effects on thoughts (*F*_3,356_=1.409; *P*=.24) and emotions (*F*_3,356_=1.850; *P*=.14) did not differ per week, the effect on body differed per week (*F*_3,356_=2.780; *P*=.04), with the strongest effect on body occurring during week 1 (comparison with week 2: –1.911, 95% CI –3.362 to –0.456; *P*=.01; comparison with week 3: –1.578, 95% CI –3.033 to –0.123; *P*=.03; and comparison with week 4: –1.678, 95% CI –3.133 to –0.223; *P*=.02).

### Relationships Among Meditation Outcomes

**Figure 5:**
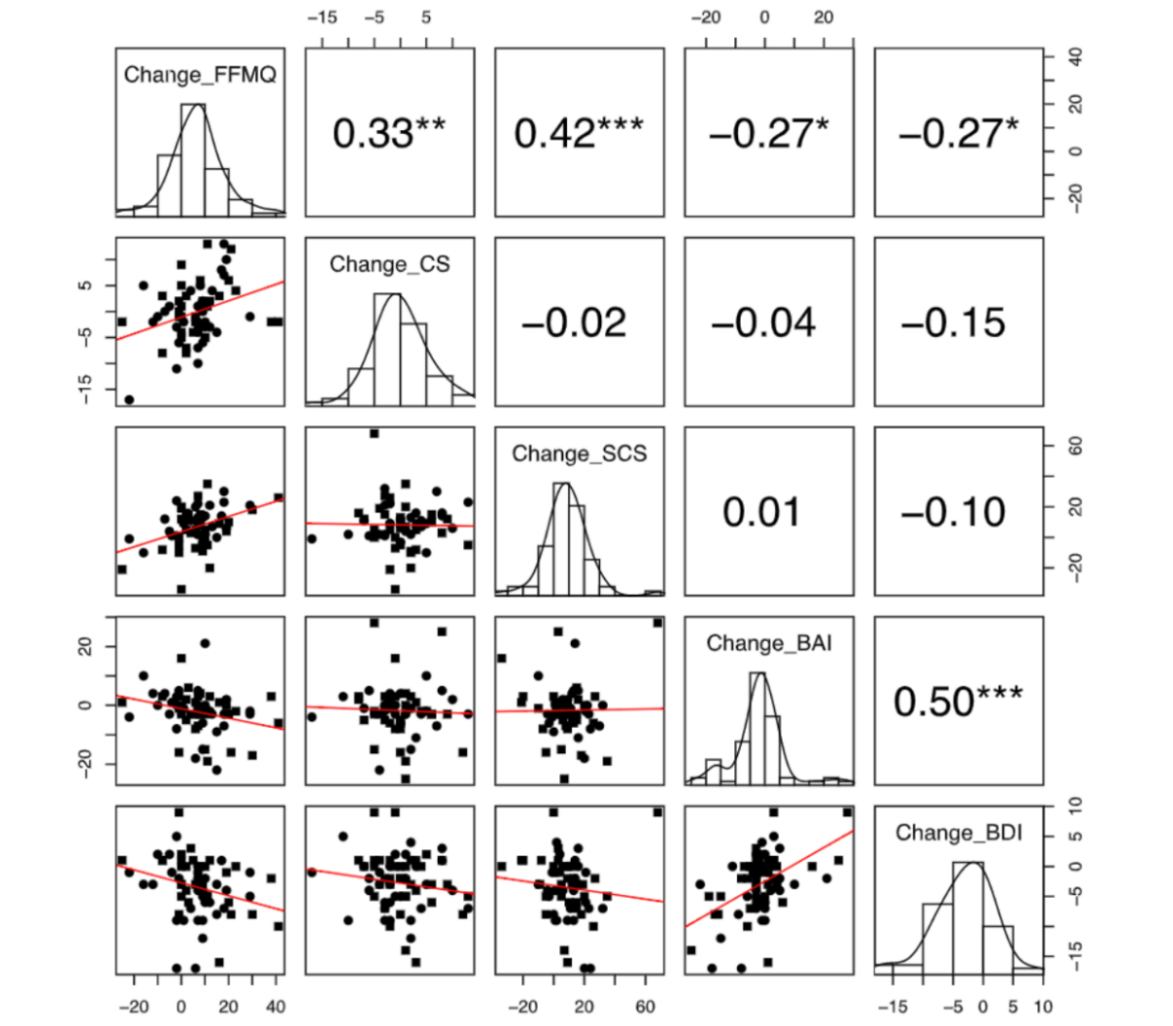
Pearson product-moment correlation coefficients (top right), histograms (diagonal), and correlation scatterplots (bottom left) demonstrating the relationships among the changes in our primary outcomes of interest from before to after the intervention. Outer edge scales represent the range of total response for each individual scale. BAI: Beck Anxiety Inventory; BDI: Beck Depression Inventory; CS: Compassion Scale; FFMQ: Five-Facet Mindfulness Questionnaire; SCS: Self-Compassion Scale. *P<.05, **P<.01, and ***P<.001.

Regarding the relationship among meditation outcomes, statistically significant correlations were found between the change in FFMQ and the change in CS (*r*=0.326; *P*=.009), SCS (*r*=0.424; *P*<.001), BAI (*r*=–0.266; *P*=.03), and BDI (*r*=–0.271; *P*=.03; Figure 5). In addition, statistically significant correlations were found between the change in MAAS and the change in SCS self-judgment (*r*=0.333; *P*=.008), BAI (*r*=–0.528; *P*<.001), and BDI (*r*=–0.314; *P*=.008; Multimedia Appendix 4). The change in neuroscience knowledge revealed only 1 significant correlation with SCS common humanity (*r*=0.287; *P*=.02). All other correlations regarding meditation outcomes are provided in Multimedia Appendix 4.

Furthermore, total daily change scores (after meditation minus before meditation) demonstrated significant correlations with the change in FFMQ (*r*=–0.251; *P*=.03), FFMQ acting with awareness (*r*=–0.235; *P*=.04), FFMQ nonreactivity (*r*=–0.239; *P*=.04), MAAS (*r*=–0.352; *P*=.002), and SCS mindfulness (*r*=–0.269; *P*=.03). In addition, total daily change score for the subscale of emotions was significantly correlated to the change in SCS (*r*=–0.258; *P*=.03). All other correlations regarding specific aspects of total daily change scores related to thoughts, emotions, and body are provided in Multimedia Appendix 4.

## Discussion

### Principal Findings

This study examined the effect of adding compassion cueing to a neuroscience-based mindfulness meditation practice on various neuropsychological outcomes. We found that although compassion cueing did not enhance our outcomes of interest, the practice of meditation enhanced mindfulness, increased self-compassion, and decreased levels of depression. Importantly, we found that those individuals who gained the most in terms of mindfulness showed the largest gains in compassion, self-compassion, and mental health. In addition, we found that although our intervention improved neuroscience knowledge, this new knowledge was not correlated to our neuropsychological outcomes of interest. Finally, we found that the acute effects of meditation were related to the long-term effects of meditation: those who gained the most in terms of acute effects benefited the most in the long term. Although our neuropsychological findings are consistent with existing literature [23,37-39], this is the first time that this novel neuroscience education and meditation program has been shown to improve psychological state. In addition, we newly show that the acute effects of this program demonstrate small yet significant correlations to the longitudinal outcomes.

### The Effects of Meditation on the Mind

Our results are consistent with existing literature demonstrating that meditation is a powerful tool to improve neuropsychological function, with the most significant positive impacts seen in self-regulatory behaviors [10,40]. Specifically, we found that this novel education-meditation intervention promoted increases in mindfulness, decreases in depression, and gains in self-compassion. However, to truly demonstrate an effect, future studies will need to include a control group that does not receive meditation and another group that does not receive the neuroscience education component. The purpose of this study, however, was to examine ways to fine-tune the effects of meditation on brain function. Together, these psychological outcomes are evidence that this 4-week mindfulness program promotes self-regulation [41,42], which includes aspects of enhanced self-awareness (ie, increased mindfulness) and emotional regulation (ie, decreased depression). Importantly, the outcome of self-compassion reflects internal flexibility, allowing an individual to be caring, kind, and nonjudgmental toward oneself, particularly during times of distress [35]. This finding indicates that this practice may be especially beneficial for populations with deficits in self-compassion, such as those with neuropsychiatric disorders (eg, major depressive disorder) [43]. Others have hypothesized that self-compassion is an important indicator of adaptive psychological function associated with mindfulness practices [14,35,44]. In fact, it is suggested that as an outcome of mindful meditation [10], self-regulation allows for greater self-compassion through meditation’s mechanisms of action at the level of the brain [45].

Similar to our work, previous studies indicate that increased mindfulness leads to improved well-being [34,46], decreased depression symptomatology [47], and reduced stress [23,38]. Well-being and stress reduction are part of preventive health measures that require active intentionality to cultivate [48]. We hypothesize, along with others, that the intentional practice of meditation may be a key way to improve well-being, with this effect being driven by the impact of meditation on self-regulatory brain processes. Research has demonstrated that enhanced self-regulation can be characterized by neuroplastic changes to the anterior cingulate cortex, insula, temporoparietal junction, frontolimbic network, and default mode network structures [49]. Therefore, future mindful meditation studies should use neuroimaging techniques to examine how these brain areas are affected and how changes in these brain structures relate to meditation-induced changes in self-regulation.

### The Effect of Teacher Cueing on Meditation Outcomes

Our novel intervention focused on the teacher’s cueing behavior (ie, functional cueing vs compassion cueing) during meditation and how cueing affected outcomes related to mindfulness, compassion, and mental health. Interestingly, we found that cueing behavior did not have a substantial impact on our outcomes of interest. Rather, we found that it is the practice of meditation itself rather than teacher behavior that affects outcomes. Similar to our work, Condon et al [50] presented mindful meditation to one group and compassion meditation to another over an 8-week period. Participants were 5 times (odds ratio 5.33) more likely after the intervention to offer compassion to others, irrespective of the type of meditation they were assigned.

This particular outcome of our study is consistent with mindful self-regulation theory [51], suggesting that mindfulness, no matter how it is practiced, has distinct and predictable outcomes that may improve health behaviors. Crane et al [25] and Baer and Wolf [52] also emphasize the importance of the behavior itself, focusing on the elements that precede it and the resulting change through the scientific field of applied behavioral analysis. This study highlights the importance of the actual practice of meditation; no matter how meditation is taught or presented, the act of engaging in meditation results in improved psychological health.

### Knowing About the Brain Is Not Necessary for Functional Improvements of the Brain

This intervention included a neuroscience education curriculum, teaching the neuroscience of 4 unique meditation techniques that focused on attention, emotional regulation, self-awareness, and self-regulation (Table 2). Our research demonstrates that participants increased their knowledge of the neuroscience that supports these meditation techniques; however, neuroscience knowledge acquisition was not related to improvements in our outcomes of interest (ie, mindfulness, self-compassion, and depression). This lack of correlation further supports the idea that practicing mindfulness meditation has a powerful effect on outcomes without the need to fully understand the process.

Although mindfulness has been examined from the perspective of neuroscience [10,53], this is the first meditation intervention designed within the context of a neuroscience curriculum. Specifically, the curriculum used in this intervention intentionally scaffolded neuroscience content and meditation skills. The curriculum built skills considering the prerequisites needed to move on to the following week: attentional control (ie, mantra) was presented first, followed by emotional control (ie, movie of the mind), self-awareness (ie, hand turning), and finally self-regulation (ie, watching the breath). Future RCTs will need to evaluate this meditation intervention with versus without the inclusion of the neuroscience curriculum.

### The Acute Effects of Meditation Correspond to the Longitudinal Outcomes of Meditation

The daily scores provided insight into how meditation acutely affected thoughts, emotions, and bodily sensations. First, we found that the most prominent acute effects occurred during the first week of the intervention, with thoughts, emotions, and body moving from active to settled from before to after the daily intervention. Subsequently, the total change scores decreased over the 20 days of the intervention; however, this change was related to the long-term beneficial effects of the intervention, that is, as the intervention progressed, participants started their daily intervention in a more settled state, with less room for improvement. This is similar to other work showing that the effects of meditation change as novice meditators gain experience [40,54-56]. Regarding the impact of each unique meditation, attentional control demonstrated the most significant acute effects, with this effect being driven by changes in bodily sensations. This may be because it was the first week of meditation. Alternatively, attentional control meditation may have had the most significant impact on acute changes at the level of the body. Although this curriculum was intentionally scaffolded, future studies may choose to alter, in terms of the sequence, the presentation of attentional control, emotional control, self-awareness, and self-regulation meditations.

Importantly, we found that the acute effects of meditation substantially related to our outcomes of interest. Specifically, those individuals who showed the largest acute benefits (from states of active to settled) showed the largest gains in various aspects of mindfulness, including acting with awareness, nonreactivity, dispositional mindfulness, and self-compassion mindfulness. In addition, those who demonstrated the largest shifts in emotion from active to settled, demonstrated the largest gains in self-compassion. This is the first study that has combined ecological momentary assessment with long-term outcomes to show that the acute effects of meditation are related to the chronic effects.

### Limitations and Future Directions

Although the results of this study are well supported by existing literature and add to existing knowledge, limitations exist. First, this study was conducted on a convenience sample that could have been more diverse. Second, because of technical issues, not all posttest data were collected from every participant, creating unequal sample sizes for some measurements. In addition, many of the predictable neuroplastic changes associated with mindful meditation may take longer to manifest than the duration of 20 days spanning over 4 weeks offered by this intervention. A future iteration of this study should be completed with each of the 4 techniques spanning 2 weeks for a total intervention duration of 8 weeks.

Meditation studies often struggle to have a solid study design and implementation methodology. Among these challenges is finding a suitable control for mindful meditation. We foresee that separating the neuroscience education from the meditation practice will create an excellent mindful meditation education control. In addition, as the intervention did not display any statistically significant differences between the control and experimental groups, future studies should include a comparison of basic cueing versus no cueing.

Finally, this study design included 5 days of meditation spread over a 7-day week. This allowed for the most flexibility from participants. If a day was missed during a traditional weekday, participants could complete their session or sessions on the weekend. Therefore, the effect of the meditation may have been lessened by this break in meditation. In addition, creating a daily routine with less flexibility may prove easier for participants. Future study designs could include (1) 20 consecutive days or (2) a longer intervention with 40 mindful meditation sessions over 8 weeks to investigate the effect of meditation dosage.

### Conclusions

This study, which used a novel neuroscience-based education–meditation program, demonstrated the behavioral importance of engaging with mindfulness meditation to optimize individual well-being through improved mindfulness, self-compassion, and depression symptomatology, suggesting enhanced self-regulation. This study additionally reinforces the idea that the benefits of meditation are independent of teacher cueing behavior. These neuropsychological changes are likely supported by neuroplastic changes associated with mindfulness meditation, but future work will need to investigate the brain-based outcomes. Finally, this study newly demonstrates that the acute effects of meditation translate into longitudinal outcomes. Future studies will need to systematically investigate the inclusive use of the neuroscience curriculum as well as test the effects of other types of teacher cueing.

## Appendix

Multimedia Appendix 1 Neuroscience knowledge questionnaire (administered both before and after the intervention).

Multimedia Appendix 2 Mean (SE of the mean) for all neuropsychological outcomes of interest for both groups both before and after the intervention. Time and time × group effects are presented. All statistically significant effects are highlighted in bold.

Multimedia Appendix 3 Ecological momentary assessment scores from before to after the daily education-meditation practice demonstrated a statistically significant change each day of the intervention. Mean change scores (pre-post assessments) are presented for each day of the intervention. Statistically significant effects are highlighted in bold.

Multimedia Appendix 4 Correlation table for all outcomes of interest, including both ecological momentary assessments and primary endpoints. All statistically significant effects are highlighted in bold.

Multimedia Appendix 5 CONSORT-eHEALTH checklist (V 1.6.2).

## Abbreviations

BAI: Beck Anxiety Inventory
BDI: Beck Depression Inventory
CS: Compassion Scale
FFMQ: Five-Facet Mindfulness Questionnaire
MAAS: Mindful Attention Awareness Scale
NKC: Neuroscience Knowledge Check
RCT: randomized controlled trial
SCS: Self-Compassion Scale
