# Supplementary material for: Effects of a Neuroscience-Based Mindfulness Meditation Program on Psychological Health: Pilot Randomized Controlled Trial
Source: JMIR Form Res. 2023 Jan 19;7:e40135. doi: 10.2196/40135 (PMC9896358; doi:10.2196/40135)
Supplement: Multimedia Appendix 1 [file formative_v7i1e40135_app1.pdf]

Neuroscience Knowledge Check

- ➔ Choose the best answer to each of the following questions.
- ➔ You may only select one option per question.
- ➔ Do not look up the answers, rather tell us what you know.
- ➔ Choose “I don’t know” instead of guessing, if you do not know the answer to the question.

Neurons are

a. Groupings of cells, working together for optimal brain function
b. Single cells with many parts
c. The unit in the brain responsible for cleaning and maintaining the brain
d. I don’t know

The synapse is

a. Where neurons join together
b. Marked with clear boundaries
c. The space between neurons
d. I don’t know

Brain regions

a. Differentiate brain function
b. Usually work in isolation
c. Control every single function of the body
d. I don’t know

The network of neurons in the brain

a. Creates the ability to perform complicated tasks in the body
b. Is limited to brain regions that touch each other
c. Requires neurons to actually touch each other
d. I don’t know

Attention in the brain, is controlled by

a. The amygdala and hypothalamus
b. The will of an individual
c. The Prefrontal Cortex, Anterior Cingulate Cortex, and Striatum
d. I don’t know

Attention requires you to

a. Resist distractions and sustain focus
b. Only resist distractions
c. Release decisions over to automatic function
d. I don’t know

Mental Conflicts arise when

a. You want something you know you don’t need
b. Your brain receives conflicting messages from various brain regions
c. Your brain has more than one region active at the same time
d. I don’t know

The Anterior Cingulate Cortex

a. Works on executive functions, decision making and goal setting
b. Works on decision making, coordinating thoughts, voluntary movement
c. Works on self-regulation, empathy, and impulse control
d. I don’t know

The Prefrontal Cortex

a. Works on executive functions, decision making and goal setting
b. Works on decision making, coordinating thoughts, voluntary movement
c. Works on self-regulation, empathy, and impulse control
e. I don’t know

The Striatum

a. Works on executive functions, decision making and goal setting
b. Works on decision making, coordinating thoughts, voluntary movement
c. Works on self-regulation, empathy, and impulse control
d. I don’t know

The Amygdala is part of the

a. Cortex
b. Brainstem
c. limbic system
d. I don’t know

The amygdala primarily processes

a. Emotions of joy and happiness
b. Emotions of fear and stress
c. All emotions equally
d. I don’t know

What are feelings?

a. Emotions
b. Physical Sensations
c. Both emotions and physical sensations
d. I don't’ know

The Limbic System of the brain includes the

a. Prefrontal cortex, Anterior Cingulate Cortex and Striatum
b. Prefrontal cortex, Anterior Cingulate Cortex, Amygdala and Insula
c. Hippocampus, Hypothalamus, Amygdala and Insula
d. I don’t know

Electro-Chemical Communication specifically happens

a. Only among neurons in the same brain region
b. Only across brain regions
c. In the synapse between neurons
d. I don’t know

Emotional Regulation

a. Includes observing a reaction, then choosing to engage and alter a response
b. Is not possible
c. Requires extreme determination
d. I don’t know

The enteric immune system

a. Is isolated in the digestive system
b. Links the digestive system and the brain
c. Is the brain’s protective immune system
d. I don’t know

Networking among brain regions

a. Happens in glial cells
b. Is faster when brain regions are closer together
c. Requires brain regions to touch each other
d. I don’t know

An individual’s emotional responses are determined by

a. That individual’s previous experiences
b. The amount of neurotransmitters are in the brain at the time of stimulation
c. That individual’s intelligence
d. I don’t know

The limbic system helps regulate

a. The balance among thinking, feeling, and reacting to a situation
b. All your emotions about a situation
c. The intensity of your reaction to a situation
d. I don’t know

Proprioception connects mind and body

a. By the mind telling the body what to do
b. As the mind knows where the body is in space
c. As the mind is aware of what’s happening inside the body
d. I don’t know

Interoception connects mind and body

a. As the mind knows where the body is in space
b. As the mind is aware of what’s happening inside the body
c. By the mind telling the body what to do
d. I don’t know

The connection among brain, spinal cord, and muscles are

a. Perkinji Cells
b. Pyramidal Neurons
c. Motor Neurons
d. I don’t know

How are neurons grouped in your body?

a. Evenly through the entire body
b. More neurons closer to your brain
c. In areas where you need them most
d. I don’t know

Top Down neuroscience emphasizes

a. How the brain and automatic responses tell the body what to do
b. How the brain and body communicate with each other
c. How the body and sensory input informs the brain
d. I don’t know

Bottom Up

a. How the brain and automatic responses tell the body what to do
b. How the brain and body communicate with each other
c. How the body and sensory input informs the brain
d. I don’t know

Balancing bottom up and top down brain activity support

a. The mind-body connection
b. The body to be strong
c. The mind to be in control
d. I don’t know

Feelings are identified best by

a. Top down neuroscience
b. Bottom up neuroscience
c. Integration of top down and bottom up
d. I don't know

The Homunculus Man is a visual representation of

a. Where neurons need to be located
b. Where we have large numbers of neurons
c. How neuroscientists think
d. I don’t know

The Striatum processes information

a. About voluntary movement
b. Using purkinje cells
c. Involved in involuntary movements

d. I don’t know

When you see a threat, three common responses are

a. Fight, flight, or fantasize
b. Fight, flight or freeze
c. Freeze, flight, or fix
d. I don’t know

The sympathetic nervous system

a. Regulates responses to life threatening situations
b. Helps the body to rest, repair and digest
c. Works in isolation from all other body systems
d. I don’t know

The parasympathetic nervous system

a. Regulates life threatening responses
b. Helps the body to rest, repair and digest
c. Works in isolation from all other body systems
d. I don’t know

The Vagus Nerve

a. Controls both conscious and unconscious actions in the body
b. Only controls body organs
c. Is named for its direct path through the body
d. I don’t know

The Vagus Nerve

a. Turns off the digestive system
b. Amplifies action of the HPA Axis
c. Connects the diaphragm to conscious and unconscious actions
d. I don’t know

Intentional slow breathing

a. Only makes you think you are slowing your body systems down
b. Happens when the diaphragm moves the ribs, expanding and contracting the lungs
c. Speeds up your heart rate
d. I don’t know

The HPA Axis responds to stress,

a. Changing the way your body processes glucose through changed liver function
b. By slowing heart rate and respiration
c. When you sleep, aiding in rest and repair
d. I don’t know

Balance of the vagus nerve and the HPA Axis

a. Requires more time spent with the HPA axis dominant
b. Allows people to react to and recover from stress
c. Means people always feel neutral
d. I don’t know

When the diaphragm contracts

a. And upper chest muscles relax, it creates space in the ribs for expanding lungs
b. So does the heart, signalling the HPA Axis
c. Your abdominal muscles should also contract to maximize the size of a breath
d. I don’t know

Resiliency

a. Can be cultivated through conscious engagement of the diaphragm and vagus nerve
b. Cannot be cultivated through conscious engagement of the the diaphragm
c. Is all about emotional regulation
d. I don’t know
