# Supplementary material for: Effects of a Neuroscience-Based Mindfulness Meditation Program on Psychological Health: Pilot Randomized Controlled Trial
Source: JMIR Form Res. 2023 Jan 19;7:e40135. doi: 10.2196/40135 (PMC9896358; doi:10.2196/40135)
Supplement: Multimedia Appendix 5 [file formative_v7i1e40135_app5.pdf]

CONSORT-EHEALTH Checklist V1.6.2 Report	Manuscript Number	40135

(based on CONSORT-EHEALTH V1.6), available at [http://tinyurl.com/consort-ehealth-v1-6].

Date completed
1/13/2023 12:57:04
by
Julia Basso

Effects of a Neuroscience-Based Mindfulness Meditation Program on Psychological Health: Pilot Randomized Controlled Trial

TITLE

1a-i) Identify the mode of delivery in the title
We did not identify the mode of delivery in the program; the program can be delivered on the computer, phone, tablet or other electronic device.

1a-ii) Non-web-based components or important co-interventions in title
We addressed this using the term, "neuroscience-based mindfulness meditation program" considering we included a neuroscience education program along with mindfulness meditation.

1a-iii) Primary condition or target group in the title
We did not include the primary condition or target group as we conducted this study in a healthy adult population.

ABSTRACT

1b-i) Key features/functionalities/components of the intervention and comparator in the METHODS section of the ABSTRACT
"Participants (n=89) were randomized to receive either functional cueing (control group) or compassion cueing (experimental group) and engaged with five 10-minute meditation sessions a week for 4 weeks."

1b-ii) Level of human involvement in the METHODS section of the ABSTRACT
No human involvement occurred. "All intervention sessions were administered through digital presentation."

1b-iii) Open vs. closed, web-based (self-assessment) vs. face-to-face assessments in the METHODS section of the ABSTRACT
"All participants completed ecological momentary assessments before and after the daily intervention, as well as pre- and postintervention questionnaires."

1b-iv) RESULTS section in abstract must contain use data
The number of participants is mentioned in the methods section of the abstract. "Participants demonstrated significant benefits over time, including increased mindfulness and self-compassion, decreased depression, and gains in neuroscience content (all P<.001); however, no significant between-group differences were found. Daily scores from each day of the intervention showed a statistically significant shift from active toward settled."

1b-v) CONCLUSIONS/DISCUSSION in abstract for negative trials
We mention negative time*group findings as: "Our findings demonstrate the behavioral importance of engaging with mindfulness meditation and reinforce the idea that the benefits of meditation are independent of teacher cueing behavior."

INTRODUCTION

2a-i) Problem and the type of system/solution
"In this single-blind randomized controlled trial (RCT), we explored how teachers of mindfulness use their words and curriculum to guide and instruct meditation to optimize
psychological outcomes, including mindfulness, compassion, self-compassion, and mental health." We also note, "Understanding the impact of the curricular implementation of
meditation will help to fill the gap in best practices and competencies for inclusion in future mindfulness interventions."

2a-ii) Scientific background, rationale: What is known about the (type of) system
"A host of literature has examined the effects of meditation on a range of psychological factors and brain outcomes, with multiple systematic reviews and meta-analyses published in this field...Although the size of the scientific literature supporting the benefits of meditation is increasing, it is currently unknown."

Does your paper address CONSORT subitem 2b?
"We hypothesized that there would be a statistically significant correlation between the gains in neuroscience knowledge and our psychological outcomes of interest. Before and after meditation, daily check-ins provided information about the acute effects of meditation on thoughts, emotions, and bodily sensations. We hypothesized that larger acute gains would correlate to larger long-term gains from meditation, which is yet another unexplored area of research."

METHODS

3a) CONSORT: Description of trial design (such as parallel, factorial) including allocation ratio
"This study was a 4-week RCT, with participants randomized to receive either meditation with functional cues (control group) or meditation with self-compassion cues (experimental group)."

3b) CONSORT: Important changes to methods after trial commencement (such as eligibility criteria), with reasons
There were no changes to methods after trial commencement.

3b-i) Bug fixes, Downtimes, Content Changes
There were no bug fixes, downtimes, or content changes during the trial.

4a) CONSORT: Eligibility criteria for participants
"To be eligible for the study, participants were required to be aged ≥18 years and be fluent in English. The exclusion criteria were active trauma or diagnosed and untreated psychiatric
illness, which were both self-reported through an initial screening questionnaire developed by the investigators."

4a-i) Computer / Internet literacy
We did not include this language in the manuscript.

4a-ii) Open vs. closed, web-based vs. face-to-face assessments:
"Recruitment was conducted using social media platforms, other web-based forums, and through in-person announcements by Virginia Tech faculty members who were teaching related content (ie, mindfulness and meditation)."

4a-iii) Information giving during recruitment
"The recruitment language included an invitation to practice meditation in the
participant's own home, learn the neuroscience supporting mindfulness meditation, and contribute to new scientific discoveries."

4b) CONSORT: Settings and locations where the data were collected
Data was collected via Qualtrics, which is mentioned several times throughout the manuscript. Example: "Clicking on the provided link brought the participants to a Qualtrics
survey with 3 daily questions."

4b-i) Report if outcomes were (self-)assessed through online questionnaires
We have included a paragraph desciption of each of the self-report measures that we have included. Example: "The Beck Anxiety Inventory (BAI) is a self-report measure of
anxiety symptoms. Questions about somatic and psychological experiences related to anxiety are included. The BAI includes 21 items for reflection on a 4-point Likert scale,
with 0 indicating "Not at all" and 3 indicating "It bothered me a lot." The total score is calculated by summing responses for each question. Results range from 0 to 21=low anxiety, 22 to 35=moderate anxiety, and ≥36=potentially concerning anxiety levels. The BAI demonstrates high internal consistency, with Cronbach α=.91 and median item correlations at r=0.56. Principal components analysis with eigenvalues >1.0 with a varimax rotation converged in 19 iterations resulted in 5 factors, which accounted for 60% of the variance."

4b-ii) Report how institutional affiliations are displayed
"The institutional review board at Virginia Tech reviewed and approved this study (IRB-20-799), and all participants completed informed consent." Participants were alerted to this information through the flyers as well as the informed consent.

5) CONSORT: Describe the interventions for each group with sufficient details to allow replication, including how and when they were actually administered

5-i) Mention names, credential, affiliations of the developers, sponsors, and owners
"The education-meditation curriculum was designed by a PhD neuroscientist and an experienced meditation teacher with >10,000 hours of teaching experience."

5-ii) Describe the history/development process
Considering this was a pilot study, this was our first permutation of this intervention.

5-iii) Revisions and updating
This was the first permutation of this intervention. We have not yet pursued further development of this intervention.

5-iv) Quality assurance methods
Quality assurance was bred into this program through back and forth discussion and permutation between, "a PhD neuroscientist and an experienced meditation teacher with
>10,000 hours of teaching experience."

5-v) Ensure replicability by publishing the source code, and/or providing screenshots/screen-capture video, and/or providing flowcharts of the algorithms used

To ensure replicability, the program will be accessible upon request.		
**5-vi) Digital preservation**		
The intervention is preserved through webservers at Virginia Tech as well as online at vimeo.com.		
**5-vii) Access**		
"Each day, participants were instructed to watch a 10-minute prerecorded education-meditation video." These videos were housed on vimeo.com and provided through Qualtrics.		
**5-viii) Mode of delivery, features/functionalities/components of the intervention and comparator, and the theoretical framework**		
"The control group received standard functional meditation directions (ie, "If you are distracted, return to the mantra."), whereas the experimental group received functional meditation directions and additional self-compassion cues (ie, "When you are distracted, remember that's okay. Try to return to the mantra."). Both groups received identical neuroscience education portions of this study."		
**5-ix) Describe use parameters**		
"The weekly schedule included a scaffolded curriculum and was the same for each of the 4 weeks. Day 1 included 7 minutes of neuroscience education and 3 minutes of meditation. Days 2, 3, and 4 included 5 minutes of neuroscience education and 5 minutes of meditation. The final day of the week was a 10-minute meditation practice (Table 1). The scaffolding of the meditation experience was specifically designed to develop meditation skills over the course of the week, building toward the culminating 10-minute practice; this design is also an example of the flipped classroom model."		
**5-x) Clarify the level of human involvement**		
Limited human involvement occurred except over email for basic communications. Once the initial Qualtrics assessment was sent, everything was generally automated.		
**5-xi) Report any prompts/reminders used**		
"If participants missed a session, reminder emails were sent to them to complete the sessions over the weekend to enable them to complete 5 sessions every 7 days."		
**5-xii) Describe any co-interventions (incl. training/support)**		
No co-interventions were included in this study.		
**6a) CONSORT: Completely defined pre-specified primary and secondary outcome measures, including how and when they were assessed**		
All outcome measures were assessed under "Study Measures." Additionally, we note, " Clicking on the provided link brought the participants to a Qualtrics survey with 3 daily questions:<br>• "How are your thoughts?"<br>• "How are your feelings?"<br>• "How is your body?"<br>The questions were answered on a standard 10-point Likert scale ranging from 1="Settled" to 10="Active." This set of questions, although not validated, showed sensitivity to change<br>in response to daily interventions."		
**6a-i) Online questionnaires: describe if they were validated for online use and apply CHERRIES items to describe how the questionnaires were designed/deployed**		
We note and provide information for all validated questionnaires under "Study Measures."		
**6a-ii) Describe whether and how "use" (including intensity of use/dosage) was defined/measured/monitored**		
"Time spent watching the 10-minute video was monitored in Qualtrics with a clock feature on the embedded video page: <10 minutes meant that the participants did not complete the session, and >10 minutes suggested that they may have been distracted with other tasks or fallen asleep. Participants displaying inconsistent times more than once (3/89, 3%) were removed from the study."		
**6a-iii) Describe whether, how, and when qualitative feedback from participants was obtained**		
We did not obtain any formal qualitative feedback from participants.		
**6b) CONSORT: Any changes to trial outcomes after the trial commenced, with reasons**		
Data was collected via Qualtrics, which is mentioned several times throughout the manuscript. Example: "Clicking on the provided link brought the participants to a Qualtrics<br>survey with 3 daily questions."		
**7a) CONSORT: How sample size was determined**		
**7a-i) Describe whether and how expected attrition was taken into account when calculating the sample size**		
"An a priori power analysis was run using G*Power (version 3.1; Heinrich Heine University) to determine the appropriate number of participants to power this study. We used an F test,<br>repeated measures ANOVA, within-between interaction using an effect size of 0.25, an α error probability of .0001 to correct for multiple testing, a power level of 0.08, 2 groups (functional cueing vs compassion cueing), 2 measurements (before the intervention vs after the intervention), correlation among repeated measures of 0.5, and a nonsphericity correction epsilon of 1 to determine a sample size of n=98."		
**7b) CONSORT: When applicable, explanation of any interim analyses and stopping guidelines**		
All outcome measures were assessed under "Study Measures." Additionally, we note, " Clicking on the provided link brought the participants to a Qualtrics survey with 3 daily questions:<br>• "How are your thoughts?"<br>• "How are your feelings?"<br>• "How is your body?"<br>The questions were answered on a standard 10-point Likert scale ranging from 1="Settled" to 10="Active." This set of questions, although not validated, showed sensitivity to change<br>in response to daily interventions."		
**8a) CONSORT: Method used to generate the random allocation sequence**		
"Participants were randomly assigned to the groups through a web-based random number generator."		
**8b) CONSORT: Type of randomisation; details of any restriction (such as blocking and block size)**		
Participants were randomly assigned to groups. There were no restrictions. "Participants were randomly assigned to the groups through a web-based random number generator."		
**9) CONSORT: Mechanism used to implement the random allocation sequence (such as sequentially numbered containers), describing any steps taken to conceal the sequence until interventions were assigned**		
"Participants were randomly assigned to the groups through a web-based random number generator.'		
**10) CONSORT: Who generated the random allocation sequence, who enrolled participants, and who assigned participants to interventions**		
The graduate student working on the project completed all of these items.		
**11a) CONSORT: Blinding - If done, who was blinded after assignment to interventions (for example, participants, care providers, those assessing outcomes) and how**		
**11a-i) Specify who was blinded, and who wasn't**		
"This RCT was partially blinded because the investigators knew participant group assignment; however, participants were unaware of their group assignment."		
**11a-ii) Discuss e.g., whether participants knew which intervention was the "intervention of interest" and which one was the "comparator"**		
"This RCT was partially blinded because the investigators knew participant group assignment; however, participants were unaware of their group assignment."		
**11b) CONSORT: If relevant, description of the similarity of interventions**		
"The control group received standard functional meditation directions (ie, "If you are distracted, return to the mantra."), whereas the experimental group received functional meditation directions and additional self-compassion cues (ie, "When you are distracted, remember that's okay. Try to return to the mantra."). Both groups received identical neuroscience education portions of this study."		
**12a) CONSORT: Statistical methods used to compare groups for primary and secondary outcomes**		
"Repeated measures ANOVAs with within-between interactions were conducted to examine the hypothesis that teacher behavior (ie, compassion cueing) significantly enhanced mindfulness (ie, FFMQ and MAAS), compassion (ie, CS and SCS), and mental health (ie, BAI and BDI) outcomes in the experimental group compared with the control group. We additionally used Pearson product-moment correlations to test the hypothesis that greater gains in neuroscience knowledge (ie, NKC) would be significantly associated with greater changes in mindfulness, compassion, and mental health outcomes and further that these<br>outcomes of interest would be significantly correlated to one another. Paired samples 2-tailed t tests were used to assess the acute effects of meditation from before to after each session. Repeated measures ANOVAs were used to determine differences among the types of acute changes (ie, thoughts, emotions, and body) across the 20 days of the intervention. An α value of P<.05 was used to determine statistical significance. To correct for multiple testing in a family of analyses, Bonferroni corrections were used as appropriate. SPSS software (version 27.0; IBM Corp) was used for all statistical analyses."		
**12a-i) Imputation techniques to deal with attrition / missing values**		
"Because of technical issues in randomization procedures (ie, Qualtrics was programmed to present 6 rather than 7 questionnaires), participants received 6 of 7 postintervention<br>neuropsychological assessments, resulting in different numbers of participant responses reported in the results." No imputation techniques were used.		
**12b) CONSORT: Methods for additional analyses, such as subgroup analyses and adjusted analyses**		
None beyond that provided in 12a were completed.		
RESULTS		

**13a) CONSORT: For each group, the numbers of participants who were randomly assigned, received intended treatment, and were analysed for the primary outcome**		
"Of the original 127 participants recruited for the study, 35 (39%) dropped out of the study before completion of the posttest assessment, and 3 (3%) were removed for lack of fidelity to research protocols, leaving a total of 89 participants."		
**13b) CONSORT: For each group, losses and exclusions after randomisation, together with reasons**		
"Of the original 127 participants recruited for the study, 35 (39%) dropped out of the study before completion of the posttest assessment, and 3 (3%) were removed for lack of fidelity to research protocols, leaving a total of 89 participants."		
**13b-i) Attrition diagram**		
We did not include an attrition diagram.		
**14a) CONSORT: Dates defining the periods of recruitment and follow-up**		
We did not included dates defining the periods of recruitment and follow-up.		
**14a-i) Indicate if critical “secular events” fell into the study period**		
We did not include if critical "secular events" fell in this period.		
**14b) CONSORT: Why the trial ended or was stopped (early)**		
The trial was not ended early.		
**15) CONSORT: A table showing baseline demographic and clinical characteristics for each group**		
Table 3 provides a demographic data table.		
**15-i) Report demographics associated with digital divide issues**		
All of those things are reported in Table 3.		
**16a) CONSORT: For each group, number of participants (denominator) included in each analysis and whether the analysis was by original assigned groups**		
**16-i) Report multiple “denominators” and provide definitions**		
We did not include these specifics. 89 participants completed the study.		
**16-ii) Primary analysis should be intent-to-treat**		
The results of all primary analyses are reported in "Study Measures Assessed Before and After the Intervention."		
**17a) CONSORT: For each primary and secondary outcome, results for each group, and the estimated effect size and its precision (such as 95% confidence interval)**		
All primary outcomes report information for both time and time*group effects. All effect sizes are reported. Example: "No significant interaction (time × group) effect was found for<br>any of our primary outcomes of interest (Multimedia Appendix 2). However, a significant time effect was found for mindfulness (Figure 1A; FFMQ: $F_{1,75}$=26.595; P<.001; partial η^2 =0.262), with mindfulness scores significantly increasing from before the intervention to after the intervention. This effect was driven by the FFMQ subscales of observing ($F_{1,75}$=12.680; P<.001; partial η^2 =0.145), describing ($F_{1,75}$=10.566; P=.002; partial η^2=0.123), acting with awareness ($F_{1,75}$=5.706; P=.02; partial η^2 =0.071), nonjudging ($F_{1,75}$=8.108; P=.006; partial η^2=0.098), and nonreactivity ($F_{1,75}$=24.622; P<.001; partial η^2=0.247).		
**17a-i) Presentation of process outcomes such as metrics of use and intensity of use**		
All participants completed all aspects of the intervention. Participants were removed from the study if they were non-adherent.		
**17b) CONSORT: For binary outcomes, presentation of both absolute and relative effect sizes is recommended**		
Partial eta squared is utilized as our effect size measure.		
**18) CONSORT: Results of any other analyses performed, including subgroup analyses and adjusted analyses, distinguishing pre-specified from exploratory**		
No exploratory analyses were utilized beyond what was reported.		
**18-i) Subgroup analysis of comparing only users**		
We did not conduct any subgroup analyses.		
**19) CONSORT: All important harms or unintended effects in each group**		
We did not find any unintended effects/harms from the intervention. Therefore, none are reported.		
**19-i) Include privacy breaches, technical problems**		
No privacy breaches or technical problems occurred.		
**19-ii) Include qualitative feedback from participants or observations from staff/researchers**		
No qualitative feedback was collected.		
DISCUSSION		
**20) CONSORT: Trial limitations, addressing sources of potential bias, imprecision, multiplicity of analyses**		
**20-i) Typical limitations in ehealth trials**		
"First, this study was conducted on a convenience sample that could have been more diverse. Second, because of technical issues, not all posttest data were collected from every participant, creating unequal sample sizes for some measurements. In addition, many of the predictable neuroplastic changes associated with mindful meditation may take longer to manifest than the duration of 20 days spanning over 4 weeks offered by this intervention."		
**21) CONSORT: Generalisability (external validity, applicability) of the trial findings**		
**21-i) Generalizability to other populations**		
This study was conducted in healthy adults. Future permutations could consider utilizing clinical populations such as those with depression or anxiety.		
**21-ii) Discuss if there were elements in the RCT that would be different in a routine application setting**		
We did not discuss whether elements in the RCT wold be different in a routine application setting.		
**22) CONSORT: Interpretation consistent with results, balancing benefits and harms, and considering other relevant evidence**		
**22-i) Restate study questions and summarize the answers suggested by the data, starting with primary outcomes and process outcomes (use)**		
"This study examined the effect of adding compassion cueing to a neuroscience-based mindfulness meditation practice on various neuropsychological outcomes. We found that although compassion cueing did not enhance our outcomes of interest, the practice of meditation enhanced mindfulness, increased self-compassion, and decreased levels of depression Importantly, we found that those individuals who gained the most in terms of mindfulness showed the largest gains in compassion, self-compassion, and mental health. In addition, we found that although our intervention improved neuroscience knowledge, this new knowledge was not correlated to our neuropsychological outcomes of interest. Finally, we found that the acute effects of meditation were related to the long-term effects of meditation: those who gained the most in terms of acute effects benefited the most in the long term. Although our neuropsychological findings are consistent with existing literature, this is the first time that this novel neuroscience education and meditation program has been shown to improve psychological state. In addition, we newly show that<br>the acute effects of this program demonstrate small yet significant correlations to the longitudinal outcomes."		
**22-ii) Highlight unanswered new questions, suggest future research**		
"A future iteration of this study should be completed with each of the 4 techniques spanning 2 weeks for a total intervention duration of 8 weeks...In addition, as the intervention did not display any statistically significant differences between the control and experimental groups, future studies should include a comparison of basic cueing versus no cueing."		
Other information		
**23) CONSORT: Registration number and name of trial registry**		
This trial was not registered as it was a pilot study.		
**24) CONSORT: Where the full trial protocol can be accessed, if available**		
The full trial protocol is not available.		
**25) CONSORT: Sources of funding and other support (such as supply of drugs), role of funders**		
"This work was supported by the integrated Translational Health Research Institute of Virginia (iTHRIV) Scholars Program, which is supported in part by the National Center for Advancing Translational Sciences of the National Institutes of Health (UL1TR003015<br>and KL2TR003016)...Finally, this work was supported by Virginia Tech’s Open Access Subvention Fund."		
**X26-i) Comment on ethics committee approval**		
"The institutional review board at Virginia Tech reviewed and approved this study (IRB-20-799), and all participants completed informed consent."		
**x26-ii) Outline informed consent procedures**		
"The institutional review board at Virginia Tech reviewed and approved this study (IRB-20-799), and all participants completed informed consent."		
**X26-iii) Safety and security procedures**		
All data was coded and will be kept confidential in the PIs laboratory 3 years past the date of publication.		
**X27-i) State the relation of the study team towards the system being evaluated**		
"Conflicts of Interest: None declared."		
